# Polygenic effects on brain functional endophenotype for deficit and non-deficit schizophrenia

**DOI:** 10.1038/s41537-024-00432-w

**Published:** 2024-02-16

**Authors:** Jin Fang, Yiding Lv, Yingying Xie, Xiaowei Tang, Xiaobin Zhang, Xiang Wang, Miao Yu, Chao Zhou, Wen Qin, Xiangrong Zhang

**Affiliations:** 1 Department of Geriatric Psychiatry, The Affiliated Brain Hospital of Nanjing Medical University, Nanjing, Jiangsu 210029 China; 2 Department of Radiology and Tianjin Key Laboratory of Functional Imaging, Tianjin Medical University General Hospital, Tianjin, 300052 China; 3 Affiliated WuTaiShan Hospital of Medical College of Yangzhou University, Yangzhou, Jiangsu 225003 China; 4 Medical Psychological Institute of the Second Xiangya Hospital, Central South University, Changsha, Hunan 410011 China

**Keywords:** Schizophrenia, Biomarkers

## Abstract

Deficit schizophrenia (DS) is a subtype of schizophrenia (SCZ). The polygenic effects on the neuroimaging alterations in DS still remain unknown. This study aims to calculate the polygenic risk scores for schizophrenia (PRS-SCZ) in DS, and further explores the potential associations with functional features of brain. PRS-SCZ was calculated according to the Whole Exome sequencing and Genome-wide association studies (GWAS). Resting-state fMRI, as well as biochemical features and neurocognitive data were obtained from 33 DS, 47 NDS and 41 HCs, and association studies of genetic risk with neuroimaging were performed in this sample. The analyses of amplitude of low-frequency fluctuation (ALFF), regional homogeneity (ReHo) and functional connectivity (FC) were performed to detect the functional alterations between DS and NDS. In addition, correlation analysis was used to investigate the relationships between functional features (ALFF, ReHo, FC) and PRS-SCZ. The PRS-SCZ of DS was significantly lower than that in NDS and HC. Compared to NDS, there was a significant increase in the ALFF of left inferior temporal gyrus (ITG.L) and left inferior frontal gyrus (IFG.L) and a significant decrease in the ALFF of right precuneus (PCUN.R) and ReHo of right middle frontal gyrus (MFG.R) in DS. FCs were widely changed between DS and NDS, mainly concentrated in default mode network, including ITG, PCUN and angular gyrus (ANG). Correlation analysis revealed that the ALFF of left ITG, the ReHo of right middle frontal gyrus, the FC value between insula and ANG, left ITG and right corpus callosum, left ITG and right PCUN, as well as the scores of Trail Making Test-B, were associated with PRS-SCZ in DS. The present study demonstrated the differential polygenic effects on functional changes of brain in DS and NDS, providing a potential neuroimaging-genetic perspective for the pathogenesis of schizophrenia.

## Introduction

Schizophrenia (SCZ) is a chronic and debilitating mental disease with high heritability. Patients with SCZ were characterized by a constellation of symptoms including hallucinations, delusions, negative mood and cognitive impairments. Previous studies have reported the lifetime prevalence of SCZ was 11.9 per 1000^1,2^. However, despite extensive research, the pathogenesis of SCZ remains unclear.

Numerous studies have revealed the potential key role of genetic alterations in SCZ. Genome-wide association studies (GWAS) have contributed to the analysis of the genetic architecture of mental disorders, identifying risk loci strongly associated with SCZ. Previous studies have demonstrated numerous risk loci single-nucleotide polymorphisms (SNPs) that were associated with clinical symptoms and antipsychotic response^3,4^. However, GWAS studies were limited by the small effect sizes of one or several SNPs, rendering them insufficient for a comprehensive understanding of the inheritance and progression of diseases^5^. To address this limitation, researchers have introduced the concept of the Polygenic Risk Score (PRS), which combines all significant risk loci identified by GWAS under a certain threshold, providing a more comprehensive measure of genetic risk for SCZ^6^. The development of PRS was facilitated to better explore the effects of genetic liability and identify more homogeneous phenotypes of SCZ. To calculate a SCZ risk score for each individual, PRS-SCZ can use the significant risk loci^7^. The potential clinical application of PRS as a tool for distinguishing SCZ is supported by its genetic predictive power^8^.

Neuroimaging studies have identified a multitude of biomarkers for SCZ. Specifically, these studies have found that patients with SCZ showed abnormal amplitude of low-frequency fluctuation (ALFF) in specific brain regions such as inferior temporal gyrus (ITG), inferior frontal gyrus (IFG), precuneus (PCUN), and calcarine fissure and surrounding cortex (CAL)^9–11^. Additionally, studies have confirmed that SCZ patients exhibit increased regional homogeneity (ReHo) values in medial prefrontal cortex and decreased ReHo values in parietal lobule and precentral lobule (PreCG)^12^. Furthermore, SCZ exhibited abnormal functional connectivity (FC) of cognitive control networks^13^. Notably, associations between decreased gray matter volumes (GMV) and lower functional connectivity (FCs) in brain networks with PRS in SCZ have been reported. These findings highlight the potential links between PRS and neuroimaging results^14,15^.

Deficit schizophrenia (DS) is a clinically homogeneous subtype of SCZ characterized by persistent and primary negative symptoms^16,17^. In contrast to non-deficit schizophrenia (NDS), DS exhibited significant differences in various biologically relevant factors, risk factors, etiologic factors, and treatment response^18^. Likewise, previous researches have demonstrated specific genetic and neuroimaging alterations in DS. For example, individuals with DS demonstrated a specific abnormality of peripheral *MMP9* expression and peripheral *CXCL1* DNA methylation^19,20^. Resting-state fMRI investigations have revealed specific alterations within the salience network (SN) that are associated with impaired neurocognition and enhanced functional integration in DS^21,22^. Researchers have identified PRS-SCZ associated with multi-site brain volumes. Negative associations were revealed between PRS-SCZ and the gray matter volume of the superior temporal gyrus, and white matter volume of the superior temporal gyrus, frontal lobe, and total white matter volume in infants^23^. In another study, within the low PRS of cross psychiatric disorders (PRScross) group, negative correlations were observed between age and the functional connectivity of the right inferior frontal cortex (IFC) with the left IFC and right inferior parietal lobe. Conversely, in the high PRScross group, no correlation was found^24^. The study combined polygenic scores for psychiatric disorders with multimodal brain images, providing new insights into the mechanisms of brain aging. However, no study has detected the PRS-SCZ of patients with DS, neither explored the associations of PRS and resting-stated fMRI.

In this study, we calculated PRS-SCZ based on whole-exome sequencing results to assess an individual’s genetic risk for SCZ. Then, we explored the differences of ALFF and ReHo among DS, NDS and HCs, as well as the FC of whole brain between DS and NDS. Additionally, the correlation analyses were performed to explore the associations between PRS-SCZ and fMRI features. We hypothesized that there would be significant differences in PRS-SCZ, ALFF, ReHo, and FC between DS, NDS, and HC. In addition, PRS-SCZ was significantly correlated with resting-state fMRI features in DS.

## Methods

### Base and target samples

Calculating PRS-SCZ by PRSice (https://www. PRSice.info) requires base samples and target samples. The base sample was employed to ascertain the effect size of genetic variation associated with disease at a predefined *P* threshold. And then, the PRS-SCZ was computed for each individual in the target sample to evaluate the overall genetic susceptibility to SCZ. In this study, the Psychiatric Genomics Consortium’s (PGC) GWAS data were utilized as the base sample for PRS-SCZ calculations, this analysis by the Schizophrenia Working Group of Psychiatric Genomics Consortium was performed on 22778 people with schizophrenia and 35362 controls (Schizophrenia Group of the PGC, 2019)^7^. The target sample consisted of 120 HCs, 81 patients with DS and 91 patients with NDS, all recruited from the psychiatric rehabilitation unit of Yangzhou Wutaishan Hospital, Jiangsu Province, China. Antipsychotic medication (Chlorpromazine) dosage was calculated for each patient^25^.The patients were diagnosed with SCZ by three experienced psychiatrists based on the Diagnostic and Statistical Manual of Mental Disorders, Fourth Edition (DSM-IV) and proven based on the Chinese version of the Structured Clinical Interview for DSM-IV (SCID-I) (First MB, 1996). The inclusion criteria were: (1) right-handed Chinese Han patients not in the base sample, (2) age from 20 to 70 years, (3) stable psychotic symptoms and antipsychotic medication for over a year. Patients with previous head trauma, mental retardation, substance abuse, or electroconvulsive therapy were excluded in this study. The patients were then divided into DS and NDS, based on the Chinese version of the Schedule for the Deficit Syndrome (SDS)^26^. Patients were considered to have DS if they had two of the following symptoms: diminished emotional range, curbing of interests, restricted affect, poverty of speech, diminished social drive, and diminished sense of purpose, which had been at least moderately severe, persistent over 1 year and not caused by secondary source like depression, paranoia, depression, paranoia, or anxiety. Inclusion criteria for healthy controls were: (1) being from the same region as the patient, (2) matching hand use habits, age and sex to the patient group, (3) healthy controls with organic brain disease or severe head trauma as well as a history of personal or family psychiatric disorder would be excluded. All participants or their guardians signed informed consent after describing the whole study to them. This study was approved by the Institutional Ethical Committee for clinical research of Zhongda Hospital Affiliated to Southeast University.

### Genotyping

For the 292 subjects, the genome-wide single-nucleotide polymorphisms (SNPs) were genotyped using the Agilent V6.0 Bead chip.

### Quality control

At the individual level, subjects would be excluded in these situations: (1) missing genotyping rate >0.05, (2) possible relative relationships, (3) as the base sample, (4) being Asian population outliers identified by multidimensional scaling (MDS). At the SNP level, SNPs with a missing call rate >0.1, minor allele frequency (MAF) < 0.01, deviation from Hardy-Weinberg equilibrium (HWE, *P* < 1e-5) would be excluded.

### Polygenic risk scores calculation

**Fig. 1:**
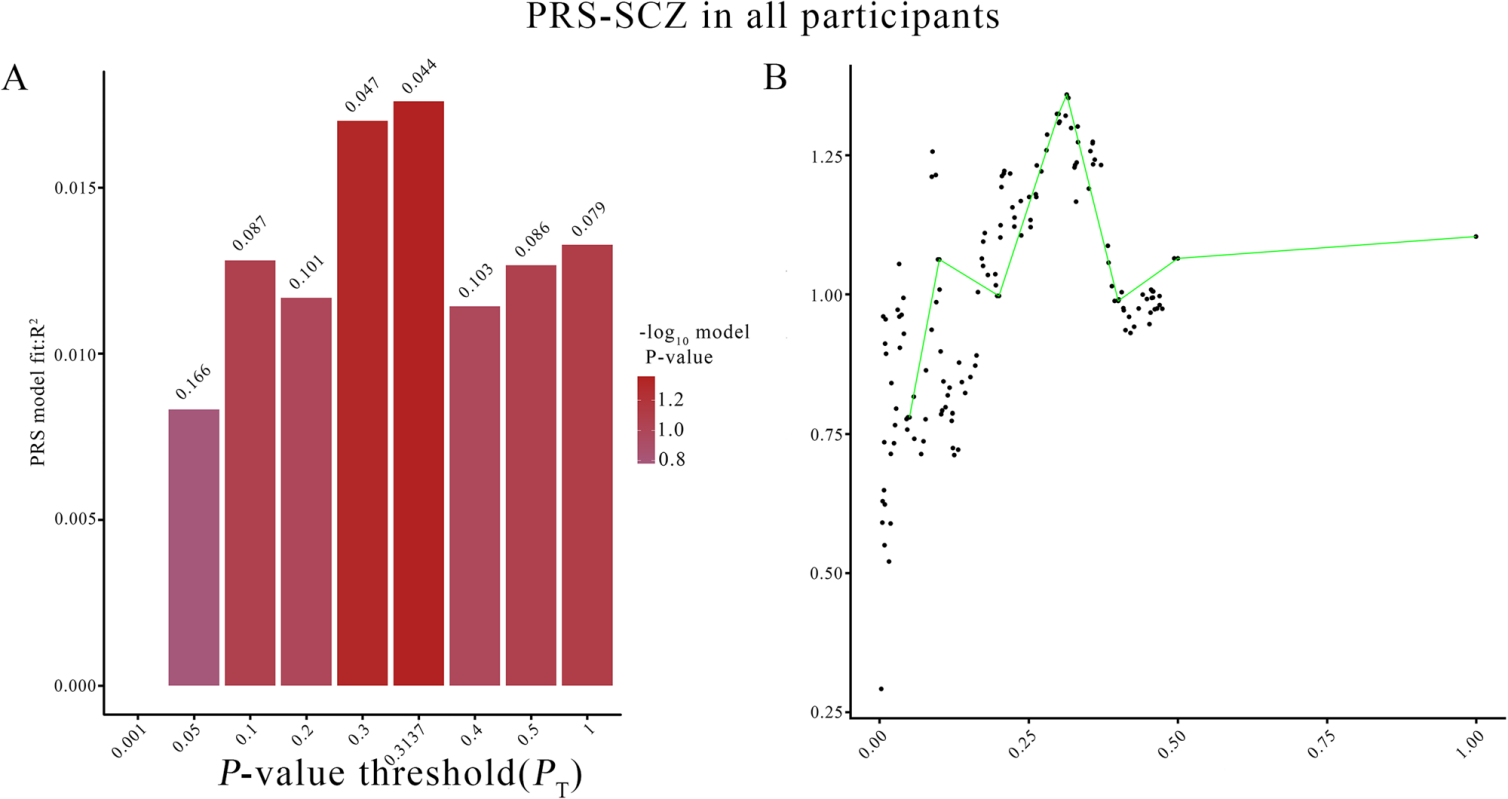
The fitting degree of PRS model under different *p*-value thresholds. PRS-SCZ, polygenetic risk score of schizophrenia. **A**: *y*-axis was the coefficient of determination (Nagelkerke’s R^2^) of the PRS model, *x*-axis was *P*-value threshold, the bar chart shows the most appropriate PRS scores calculated under nine different *p* thresholds. **B**: *y*-axis represents PRS model fit: *P* value (-log_10_), *x*-axis is the continuous *p* values threshold, from 0 to 0.5, and *p* values of 1, the point plot indicates where is the *p* value of the best PRS model to be found in the case of continuous *p* value threshold.

PRS-SCZ represents an individual’s overall genetic risk of having SCZ^27^, and higher PRS-SCZ implies that individuals are at higher genetic risk of SCZ. The SNPs of the PGC GWAS data were clumped on the basis of linkage disequilibrium (LD) and association *p* values on the PLINK (https://www.cog-genomics.org/plink/1.9/) using 1000 Genomes phase III EAS reference (https://alkesgroup.broadinstitute.org/LDSCORE/). These SNPs were clumped in 2 rounds; round 1 with the default parameters (physical distance threshold 250 kb and linkage disequilibrium *r*^2^ ≥ 0.25) using PLINK version 1.90, and round 2 was performed on the PRSice software with a physical distance threshold of 250 kb and linkage disequilibrium *r*^2^ ≥ 0.1^28^. PRS-SCZ was calculated for each subject based on the summary statistics (effect alleles and odds ratios), the summary statistics was derived from the clumped PGC GWAS results^7^. Odds ratios in the summary statistics were log-converted to beta values. The PRS-SCZ calculation was carried out in the PRSice software to generate 10,000 PRS values for *P*_T_ ranging from 0.0001 to 0.5 with an increment of 0.00005. We could identify the best *P*_T_ values for generating the best-fit model (Nagelkerk’s pseudo-*r*^2^) in the target sample through Logistic regressions (Fig. 1).

### Biochemical index and neurocognitive assessments

In order to further improve the characterization, blood was drawn from each subject for biochemical features testing, such as triglycerides (TG), cholesterol (CHO), high-density lipoprotein (HDL), low-density lipoprotein (LDL), glutamic acid (GLU), brain-derived neurotrophic factor (BDNF), glial-derived neurotrophic factor (GDNF) and prolactin (PRL). The neurocognitive of each participant with SCZ was assessed by a series of classical neurocognitive tests, such as: the Brief Psychiatric Rating Scale (BPRS), Controlled Oral Word Association Test (COWAT), Trail Making Test-A, B (TMT-A, B), Stroop Color-Word Test (SCWT), and Spatial Processing (Block design). These neurocognitive scales comprehensively assess each participant from four aspects of rational cognition: cognitive flexibility (including TMT-B and Stroop interference), sustained attention (including TMT-A, Stroop words and Stroop colors), ideation fluency (including COWAT), and visuospatial memory (including Spatial processing test)^29,30^. Based on the mean and standard deviation of HC, the raw score of each scale is converted to a Z-score, and the composite score of each cognitive aspect is calculated as the mean of the Z-score of the relevant test.

### Image acquisition

Subjects were scanned with a 3.0 T MR system (GE HDx, Chicago, Illinois) with an 8-channel phased array head coil in the Subei Hospital in Yangzhou, Jiangsu Province, China. Respecting the principle of voluntariness, only some participants collected image data, and the HC group had 41 subjects, DS group had 33 subjects, NDS group had 47 subjects. Using foam pads and earplugs to minimize head motion and scanner noise. We also asked each participant to close their eyes, stay awake and reduce head movement during the scan. Images were collected using a gradient recalled echo echo-planar imaging (GRE-EPI) sequence: TR = 2000 ms, TE = 25 ms, FOV = 24 cm × 24 cm, flip angle = 90°, matrix = 64 × 64, slices = 35, slice thickness = 4 mm, no gap, voxel size = 4 × 4 × 4 mm^3^, 240 volumes, scan time 8 min.

### Preprocessing

Image data preprocessing was conducted using the Data Processing Assistant for Resting-State fMRI (DPARSF, V5.1, http://www.restfmri.net), a program based on SPM 8 (http://www.fil.ion.ucl.ac.uk/spm/software/spm8) in MATLAB 2016b (http://www.mathworks.com/products/matlab/). Initially, the first 10 scanned volumes would be discarded to allow for the magnetization equilibrium. We then corrected for slice timing and head motion. Subsequently, the resulting function images were used 12-parameter affine to transform and nonlinear deformation were transformed into the standard EPI template in the Montreal Neurological Institute (MNI) space and the data were resampled to 3 mm isotropic voxels. Linear detrending and temporal band-pass filtering (0.01−0.08 Hz) were performed to decrease the effects of low-frequency drifts and high-frequency physiological noise. Finally, images were spatially smoothed with a Gaussian kernel of 4 × 4 × 4 mm^3^ full-width at half-maximum. There was no significant difference in the amplitude of head movement among the three groups (*p* > 0.05).

### ALFF and ReHo analysis

The ALFF and ReHo were analyzed by the Data Processing & Analysis for Brain imaging (DPABI V7.0) (http://rfmri.org/dpabi). Specifically, the filtered times series of whole-brain signal to transform it into frequency domain power spectrum. Then, on the power spectrum at the range of 0.01−0.08 Hz, calculating the root-mean-square as the ALFF. ALFF value of each voxel was normalized to the Z value (zALFF) to remove the differences of brain ALFF in the overall level between participants. We then got a zALFF map and individual zALFF map were divided into 90 cerebral regions by the Anatomical Automatic Labeling (AAL) atlas. AS for the ReHo, the ReHo maps of participants were produced by calculating Kendall’s coefficient of concordance (KCC) of the times series of this voxel its 26 nearest neighbors. To reduce the difference of brain ReHo in the general level among participants, the ReHo value of each voxel was transformed into the Z value (zReHo). Subsequently, we acquired individual zReHo maps and zReHo map were segmented into 90 cerebral regions by the AAL. The mean of the ReHo indexes in each region serves as the ALFF index for that region. The two-sample *t* test was performed to find the different brain regions of ALFF and ReHo between groups, with age and education as the covariates. Permutation tests with threshold free cluster enhancement (TFCE) was applied for comparison (number of permutations = 1000). The TFCE significance threshold was set at <0.05.

### Functional connectivity network analysis

The DPABI was used to perform FC network analyses. In this study, three differential zALFF regions between DS and NDS as seed points to make FCs network map of every seed point throughout the brain. To be specific, the three seed points included the left ITG (coordinate: −42, 12, −45), the left IFG (coordinate: −30, 9, −18), and the right PCUN (coordinate: 12, −51, 27). In the same way, only one differential zReHo region between DS and NDS as seed points to make FC network map of every seed point throughout the brain. Specifically, the seed point was the right middle frontal gyrus (MFG, coordinate: 45, 18, 24). Also, by reviewing the literatures we found an expanded number of seed points in specific brain regions of the SCZ, including the right posterior central gyrus (PoCG, coordinate: 41, −75, 53, radius: 6 mm), where decreased connectivity is associated with poorer cognitive and executive function; the right Rolandic operculum (ROL, coordinate: 53, −6, 15, radius: 6 mm), a region where gray matter abnormalities are associated with social cognitive function; the left insula (INS, coordinate: −35, 7, 3, radius: 6 mm), the right INS (INS, coordinate: −35, 7, 3, radius: 6 mm), where altered activities were associated with cognitive function^31–34^. Two-sample *T* tests were employed to identify differences in FCs across the whole brain between DS and NDS groups, with age and education as covariates. The significance level was set at voxel *p* < 0.01, corrected using Gaussian random field (GRF) correction for cluster *p* < 0.01.

### Statistical analysis

Statistical analysis was carried out using the Statistical Package for the Social Sciences (SPSS) software version 21.0 (IBM, Armonk, New York). Demographic differences among groups were examined through analysis of variance (ANOVA) with the least-significant difference (LSD) correction. To compare the PRS-SCZ among groups, analysis of covariance (ANCOVA) was employed, with age and years of education as covariates. The analysis was also corrected with LSD. Receiver Operating Characteristic (ROC) curves were utilized to distinguish DS, NDS, and HC groups. Pearson correlation analysis was applied to calculate the correlation coefficient (*r*-value) for various features, including fMRI features, biochemical indexes, neurocognitive assessments, and PRS-SCZ. Statistically significant correlations were identified at a significance level of *p* ≤ 0.05, and the results were further subjected to False Discovery Rate (FDR) correction. Finally, linear regression was employed to examine indicators displaying a linear relationship with PRS-SCZ.

## Results

### Demographic and neurocognitive assessments

The results of demographic data were shown in Table 1. There was no significant difference between patients and HCs in age (*F* = 0.284, ANOVA *p* = 0.753) and education (*F* = 2.361, ANOVA *p* = 0.096). Table S1. showed the demographic, neurocognitive assessment scores and biochemical data of the samples with both genetic and imaging data. In this subset of the sample, no significant differences in age (*p* = 0.828) and education (*p* = 0.144) among DS, NDS and HC. Moreover, the results showed that both DS and NDS group performed worser than HCs on all neurocognitive assessments, including TMT-A (s), Spatial processing (block design) (all *p* < 0.05, corrected by LSD).

**Table 1:** Demographic data of DS, NDS and HC.

Variable	DS	NDS	HC	*F/t*	*P*
	(*n* = 81)	(*n* = 91)	(*n* = 120)		
Age (years)	50.25 ± 8.369	49.87 ± 7.79	50.7 ± 7.638	0.284	0.753
Education (years)	9.08 ± 2.540	9.21 ± 1.785	9.78 ± 2.805	2.361	0.096
Age at Onset (years)	24.63 ± 7.319	23.76 ± 5.218		0.821	0.366
Duration of illness (years)	32.44 ± 8.741	30.58 ± 8.471		2.009	0.158
CPZ-equivalent daily dose (mg/day)	538.57 ± 213.338	566.43 ± 174.438		−0.911	0.364

Table 1 shows the demographic data of the whole sample. Data is the mean ± SD. DS is the deficient schizophrenia group, NDS is the group of non-defective schizophrenia patients and HC is the healthy control group. Post-hoc comparisons used the least-significant difference (LSD).

### Polygenic risk score analysis

**Fig. 2:**
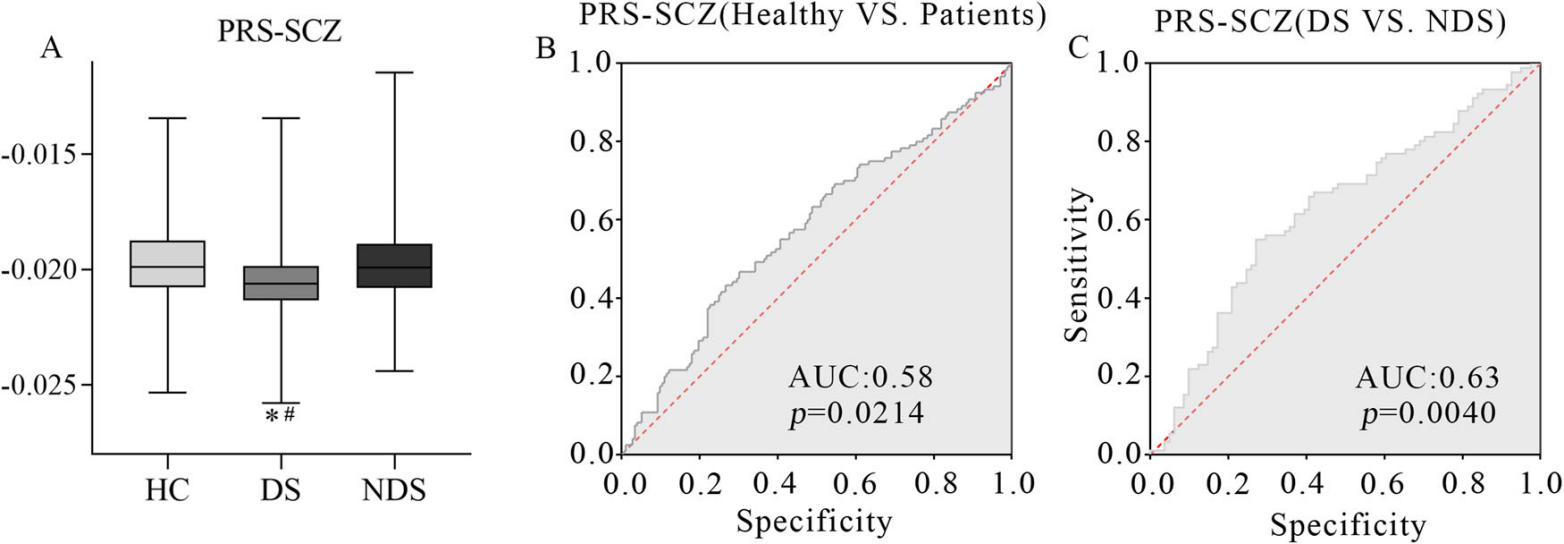
Differences in PRS-SCZ among HC, DS and NDS groups (F = 3.656, ANOVA *p* = 0.027). PRS-SCZ, polygenetic risk score of schizophrenia. **A**: *x*-axis represents three different groups: HC, DS, NDS, *y*-axis is the mean of PRS-SCZ. HC healthy control, DS deficit schizophrenia, NDS non-deficit schizophrenia. ^*^The mean of PRS-SCZ of DS is significantly different from that of HC, ^#^The mean of PRS-SCZ showed significant difference between DS and NDS groups. **B**, **C**: *x*-axis represents the specificity; *y*-axis is the sensitivity. AUC area under curve.

As shown in the Fig. 1, after excluding SNPs with a MAF < 0.1, missing call rate >0.1 or Hardy-Weinberg disequilibrium significance <0.00001, and clumping SNPs to be more significant SNPs with LD (r^2^ ≥ 0.25) within a 250 kb, 95 SNPs were included to construct the computational PRS model with the *p* value threshold of 0.3137 and an explained variance of 0.0176 (Nagelkerk’s pseudo-*r*^2^). The number of SNPs used to calculate PRSs for schizophrenia at different P thresholds is shown in Table S2. The means and standard deviations of PRS-SCZ for HC, DS and NDS are shown in Fig. 2A. The results indicated that PRS-SCZ was significantly different among the three groups (F = 3.656, ANOVA *p* = 0.027), with DS showed lower PRS-SCZ compared to NDS (*p* = 0.032**)**. The ROC analysis indicated that the area under the curve (AUC) between the patient group and the healthy control group was 0.58 (Fig. 2B, *p* = 0.0214), while the AUC between DS and NDS was 0.63 (Fig. 2C, *p* = 0.004).

### ALFF

**Fig. 3:**
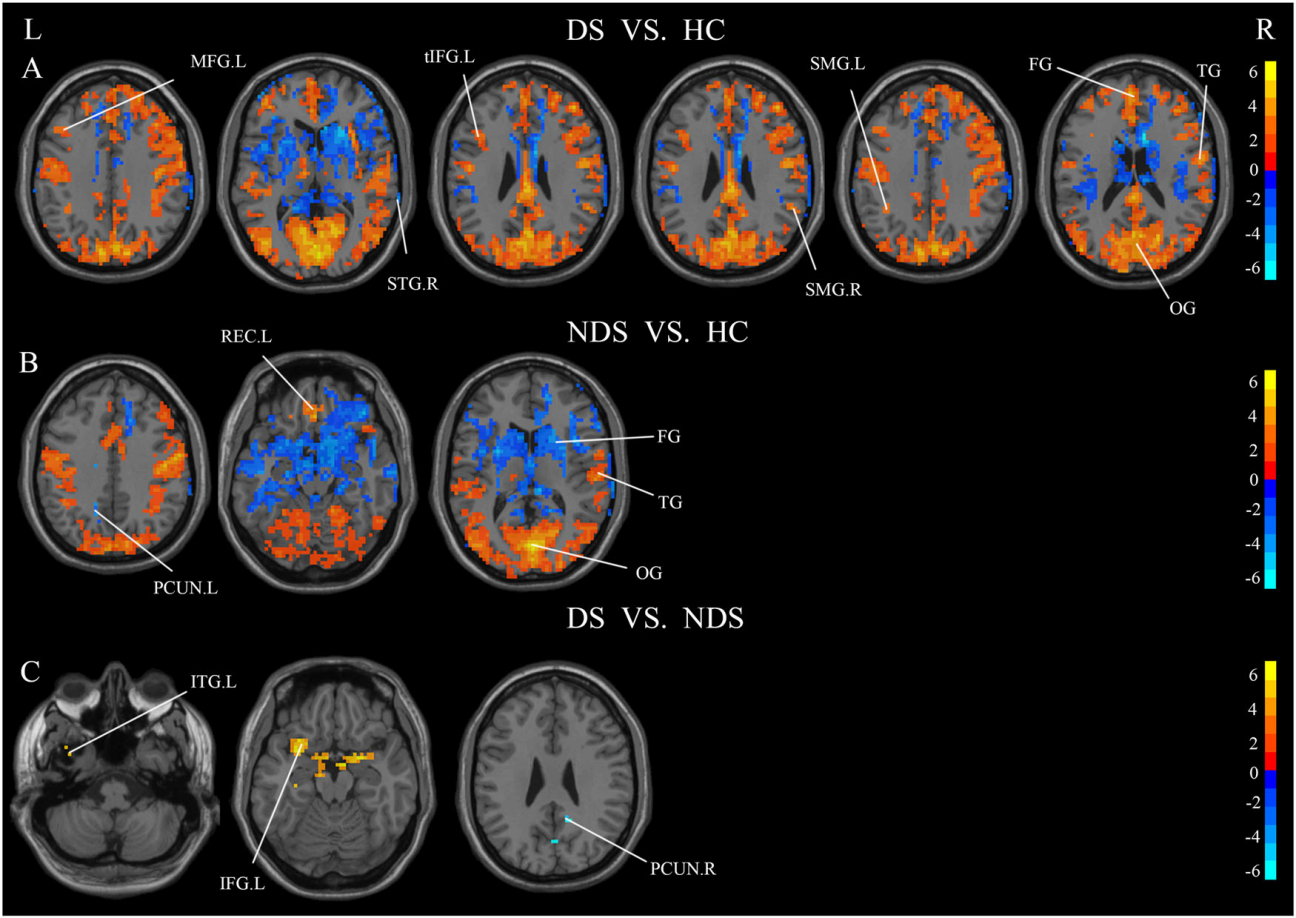
Significant Differences in ALFF values among HC, DS and NDS groups (TFCE, *p* < 0.05, GRF-corrected). Significance level was set to voxel *p* < 0.01 with Gaussian random field correction for cluster *p* < 0.01, the color bars were *t* value. HC: healthy control, DS: deficit schizophrenia, NDS: non-deficit schizophrenia. L: left, R: right. **A**: the difference in ALFF values between DS and HC, red and blue respectively represent regions where DS had higher and lower activity compared to HC. **B**: the difference in ALFF values between NDS and HC, red and blue respectively represent regions where NDS had higher and lower activity compared to HC. **C**: the difference in ALFF values between DS and NDS, red and blue respectively represent regions where DS had higher and lower activity compared to NDS.

DS and NDS patients were found to have significant abnormal ALFF in multiple brain regions when compared to HCs (Fig. 3A, B). Specifically, the ALFF in DS exhibited a noteworthy increase in the left MFG, the triangular portion of the left Inferior Frontal Gyrus, the bilateral Supramarginal Gyrus (SMG), and the brain region encompassing the Frontal, Parietal, and Occipital Lobes. Conversely, a significant decrease in ALFF was noted in the STG.R in individuals with DS (Fig. 3A). Moreover, the ALFF of the NDS exhibited a significant reduction in the PCUN.L and Frontal Lobes. Conversely, there was a notable increase in ALFF observed in the left Gyrus Rectus and the broader region encompassing the Occipital Lobe (Fig. 3B). In comparison to NDS, the ALFF of DS exhibited a significant increase in ITG.L and IFG.L, while experiencing a noteworthy decrease in PCUN.R (Fig. 3C). The results of zALFF comparisons among DS, NDS and HCs are presented in Table 2.

**Table 2:** Significant difference regions of zALFF among DS, NDS and HC.

Regions	Hemisphere	Peak MNI Coordinates			Cluster Size
		x	y	z	
DS vs HC					
Frontal Lobe/Occipital Lobe/Temporal Lobe/Limbic Lobe/Parietal Lobe	Bilateral	9	15	21	26330
Superior Temporal Gyrus	Right	72	−36	6	10
Inferior frontal gyrus, triangular part	Left	−39	18	27	30
SupraMarginal Gyrus	Right	54	−45	27	21
SupraMarginal Gyrus	Left	−39	−48	36	10
Middle Frontal Gyrus	Left	−42	24	36	19
NDS vs HC					
Frontal Lobe/Occipital Lobe/Temporal Lobe/Parietal Lobe/Limbic Lobe/Precentral Gyrus	Right	3	−81	12	21693
Gyrus rectus	Left	0	33	−18	100
Precuneus	Left	−15	−48	39	37
DS vs NDS					
Inferior Temporal Gyrus	Left	−42	12	−45	9
Inferior Frontal Gyrus	Left	−30	9	−18	211
Amygdala	Right	18	0	−12	114
Precuneus	Right	36	12	3	7

*MNI* Montreal Neurological Institute.

### ReHo

**Fig. 4:**
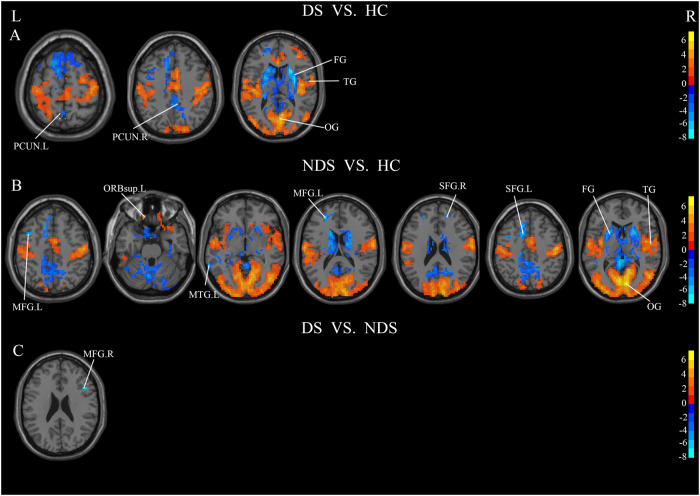
Significant Differences in ReHo values among HC, DS and NDS groups (TFCE, *p* < 0.05, GRF-corrected). Significance level was set to voxel *p* < 0.01 with Gaussian random field correction for cluster *p* < 0.01, the color bars were *t* value. HC healthy control, DS deficit schizophrenia, NDS non-deficit schizophrenia. L left, R right. **A**: differences in ReHo values between DS and HC groups, red and blue respectively represent regions where DS had higher and lower activity compared to HC. **B**: differences in ReHo values between NDS and HC groups, red and blue respectively represent regions where NDS had higher and lower activity compared to HC. **C**: differences in ReHo values between DS and NDS groups, red and blue respectively represent regions where DS had higher and lower activity compared to NDS.

Compared to HCs, there were significant differences in ReHo of various brain regions in DS and NDS patients (Fig. 4A, B). Specifically, DS demonstrated a noteworthy increase in ReHo in brain regions, including the Parietal, and Occipital Lobes. Conversely, a significant decrease in ReHo was noted in the PCUN.L, PCUN.R and Frontal Lobes (Fig. 4A). The ReHo of the NDS exhibited a significant reduction in MFG.L, MTG.L, SFG.R, SFG.L and Frontal Lobes, while experiencing a noteworthy increase in the orbital part of superior frontal gyrus, the brain region encompassing the Parietal, and Occipital Lobes (Fig. 4B). In comparison to NDS, the ReHo of DS exhibited a significant decrease in MFG.R (Fig. 4C). The results of zReHo comparisons among DS, NDS and HCs are presented in Table 3.

**Table 3:** Significant difference regions of zReHo among DS, NDS and HC.

Regions	Hemisphere	Peak MNI Coordinates			Cluster Size
		x	y	z	
DS vs HC					
Frontal Lobe/Occipital Lobe/Temporal Lobe/Parietal Lobe/Limbic Lobe	Right	21	15	12	17017
Precuneus	Right	0	−42	42	186
Precuneus	Left	−3	−54	63	10
NDS vs HC					
Occipital Lobe/Parietal Lobe/Frontal Lobe/Temporal Lobe/Cuneus/Cerebellum Posterior Lobe/Limbic Lobe/Precuneus	Bilateral	9	−81	6	14,942
Superior Frontal Gyrus, orbital part	Left	−15	42	−27	38
Middle Temporal Gyrus	Left	−66	−51	−3	138
Middle Frontal Gyrus	Left	−27	45	18	47
Superior Frontal Gyrus	Right	18	48	24	13
Superior Frontal Gyrus	Left	−12	27	45	261
Middle Frontal Gyrus	Left	−39	9	48	14
DS vs NDS					
Middle Frontal Gyrus	Right	45	18	24	18

*MNI* Montreal Neurological Institute.

### Functional connectivity

**Fig. 5:**
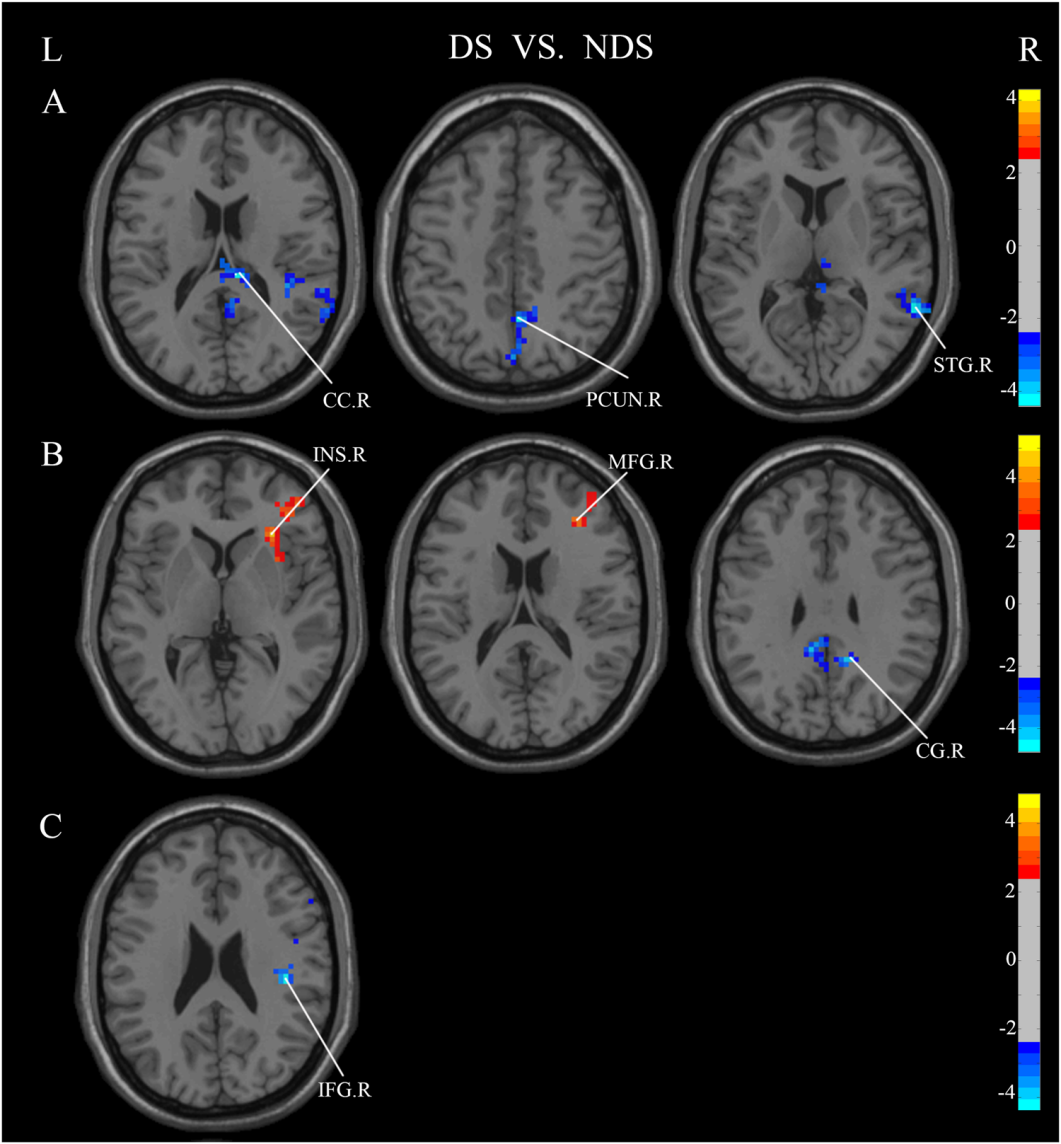
Significant Differences in functional connectivity between DS and NDS groups (GRF-corrected). Significance level was set to voxel *p* < 0.01 with Gaussian random field correction for cluster *p* < 0.01, the color bars were *t* value. DS: deficit schizophrenia, NDS: non-deficit schizophrenia. L: left, R: right. **A**: with the left inferior temporal gyrus as the seed point, the significant different functional connections between DS and NDS, **B**: with the right precuneus as the seed point, the significant different functional connections between DS and NDS, **C**: with the right middle frontal gyrus as the seed point, the significant different functional connections between DS and NDS. Red and blue respectively represent regions where DS had higher and lower strength of functional connectivity compared to NDS.

**Fig. 6:**
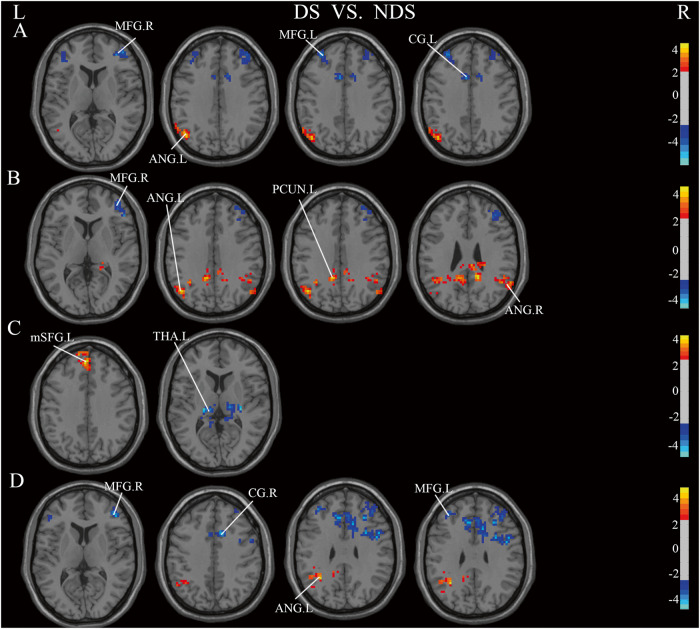
Significant Differences in functional connections between DS and NDS groups (GRF-corrected). Significance level was set to voxel *p* < 0.01 with Gaussian random field correction for cluster *p* < 0.01, the color bars were *t* value. DS: deficit schizophrenia, NDS: non-deficit schizophrenia. L: left, R: right. **A**: with the left insula as the seed point, the difference between DS and HC, **B**: with the right insula as the seed point, the difference between NDS and HC, **C**: with the right postcentral as the seed point, the difference between DS and NDS, **D**: with the right rolandic operculum as the seed point, the difference between DS and NDS. Red and blue respectively represent regions where DS had higher and lower strength of functional connectivity compared to NDS.

Analysis of FC was performed only in DS and NDS. The results of FC comparisons among DS, NDS are presented in Table S3–S10. When comparing NDS to DS, functional connectivity strengths between distinct brain regions, using the different brain regions in ALFF as seed points, the intensities of functional connectivity between ITG.L and Right Corpus Callosm, ITG.L and PCUN.R, ITG.L and STG.R, as well as PCUN.R and CG. R exhibited marked decreases in DS, Conversely, there were notable increases in functional connectivity strength observed in DS, particularly between PCUN.R and INS.R, as well as between PCUN.R and MFG.R. The different brain regions in ReHo were used as seed points, and the results suggested, DS demonstrated lower strength of functional connectivity between MFG.R and IFG.R compared to NDS (Fig. 5A–C). When employing expanded brain regions as the seed points, a marked increase in functional connectivity strength was observed within the DS between INS.L and ANG.L. Conversely, significant reductions were noted in connectivity between INS.L and MFG.R, INS.L and MFG.L, as well as INS.L and CG. L (Fig. 6A). When INS.R was used as the seed point, a significant reduction in functional connectivity strength was observed within the DS between INS.R and MFG.R. Conversely, there was a notable increase in functional connectivity strength between INS.R and ANG.R, INS.R and PCUN.L, as well as INS.R and ANG.L (Fig. 6B). In DS, the functional connectivity strength exhibited a significant decrease between PoCG.R and THA.L, ROL.R and MFG.R, ROL.R and CG.R, ROL.R and MFG.L, while it showed a notable increase between PoCG.R and mSFG.L, ROL.R and ANG.L (Fig. 6C, D).

### Correlation analysis

In DS, the value of ALFF in left ITG was negatively correlated with PRS-SCZ (*r* = −0.3916, *p* = 0.0242, Fig. S2B), and the significance was re-evaluated after FDR correction. However, the correlation did not remain statistically significant after correction. Similarly, the value of ReHo in right MFG was positively correlated with PRS-SCZ (*r* = 0.3642, *p* = 0.0372, Fig. S2A), but this correlation did not survive FDR correction. Furthermore, the PRS-SCZ of DS was initially found to be positively correlated with the strength of functional connectivity (FC) between the left ITG and the right corpus callosum (*r* = 0.4331, *p* = 0.0118, Fig. S2E), and between the left ITG and the right PCUN (*r* = 0.3820, *p* = 0.0282, Fig. S2F). However, these correlations did not withstand FDR correction (adjusted *p* > 0.05). Additionally, the FC between the right INS and the left ANG (*r* = −0.3800, *p* = 0.0291, Fig. S2C), as well as between the right INS and the right ANG (*r* = −0.3733, *p* = 0.0324, Fig. S2D), initially showed negative correlations with PRS-SCZ in DS. PRS-SCZ of DS was positively correlated with the scores of TMT-B (*r* = 0.3485, *p* = 0.0469, Fig. S1A). These correlations were not found to be statistically significant after FDR correction. In addition, In the NDS, PRS-SCZ was positively correlated with the value of FC between left INS and left MFG (*r* = 0.2978, *p* = 0.0421, Fig. S1B). This correlation did not withstand FDR correction. PRS-SCZ and blood concentrations of BDNF (*r* = −0.4847, *p* = 0.0006, Fig. S1C, FDR corrected) and HDL (*r* = −0.3910, *p* = 0.0066, Fig. S1D, FDR corrected) were negatively correlated in NDS. *P* values for correlation analysis are uncorrected. In the linear regression analysis, PRS-SCZ of DS was linearly correlated with TMT-B scores (β = 0.384, *p* = 0.009) and value of FC between left ITG and right PCUN (β = 0.328, *p* = 0.049). The PRS-SCZ of NDS and the blood concentration of BDNF was linearly correlated (β = −0.436, *p* = 0.001).

## Discussion

The study showed significant differences in PRS-SCZ among the DS, NDS and HCs, and further demonstrated the associations between altered functional features and PRS-SCZ. However, it is important to note that this correlation did not withstand FDR correction. Despite the lack of significance post FDR correction, it is important to note and discuss these trends in the context of our findings. Specifically, DS had the lowest genetic risk of SCZ, and the altered ALFF in ITG and abnormal FC of INS and ANG in DS was negatively associated with PRS-SCZ. To our knowledge, this is the first study to explore PRS-SCZ and related specific resting-state fMRI features in DS and NDS.

In the current study, the patients with deficit schizophrenia exhibited the most abnormal PRS-SCZ. Additionally, the individuals with DS were recognized for having the most severe negative symptoms, as indicated by their neurocognitive assessments. It is noteworthy that a connection between PRS-SCZ and the severity of negative symptoms, cognitive impairments, and the course of schizophrenia has been established in previous studies^35^. Our findings were consistent with prior research, which reported more pronounced negative symptoms in subjects with higher PRSs for schizophrenia^36^. Indeed, our results affirm this relationship, demonstrating that individuals deviating more from the normal PRS-SCZ exhibit more severe negative symptoms. Furthermore, it corroborates the clinical observation that individuals with DS are less frequently encountered than those without DS in clinical settings. A meta-analysis, conducted by Roy et al., revealed that the prevalence of DS was 28.7% within the entire schizophrenia population, and it did not exceed 50%^37^. Notably, this prevalence appeared to be associated with male gender and was seemingly unaffected by diagnostic labels or illness duration. Furthermore, we employed PRS-SCZ as a distinct variable to differentiate between DS and NDS, achieving an AUC of 0.63. It’s interesting to note that Ripke et al. also employed PRS-SCZ to predict schizophrenia and attained a similar AUC of 0.65^38^. However, when we used PRS-SCZ as a standalone variable to distinguish between SCZ and HCs, the AUC remained relatively modest. This could be attributed to the multifactorial nature of schizophrenia development, influenced by various factors such as environment and emotional factors. Additionally, the accuracy of PRS-SCZ might be constrained by the number of confirmed risk loci for schizophrenia. Future research endeavors could seek to enhance classification accuracy by integrating genetic factors with neuroimaging, cognitive, and biochemical variables.

Furthermore, our study revealed a positive correlation between ReHo in the right MFG and PRS-SCZ in individuals with DS. These findings gain significance in light of prior research that has also reported a positive association between PRS-SCZ and right frontal gyrification in individuals at high risk of developing schizophrenia^39^. Moreover, evidence suggested that the functional activity of the right MFG is diminished in individuals with schizophrenia and associated with lower levels of verbal fluency^40^, which indicated that the functional activity of the right middle frontal gyrus may directly reflect the cognitive function of patients. The MFG encompasses both the caudal and nasal regions, with the latter housing a portion of the dorsolateral prefrontal cortex (DLPFC), known for its role in working memory and executive functions^41^. The DLPFC served as a pivotal node in the central executive network (CEN), playing a crucial role in working memory and cognitive functions^42^^,^^43^. Our study offered insight into the potential influence of genetic factors on the development of psychiatric disorders, as they may exert their effects by modulating the functioning of the central executive network in DS.

Meanwhile, the FC of the INS and ANG was correlated with PRS-SCZ in the DS. Previous studies have found a positive correlation between PRS-SCZ and the strength of the FC between the INS and ANG in healthy populations^44^. However, we found the PRS-SCZ was negatively correlated with the FC of right INS and left ANG in DS. Therefore, more large-sample studies are needed in the future to confirm this association. The ANG is an important node in the DMN, involving cognition, attention, memory extraction and other important functions and the INS was a part of salience network (SN)^45^. Interestingly, studies have found that the interaction between the DMN and CEN was abnormally dependent on the activity of the SN, and the impaired activity in these regions was associated with psychosis in schizophrenia^46^. Our study indicates that these abnormal interactions may be modulated by genetic factors in DS. TMT-B was commonly used to measure executive function in the brain^47^, and we found the scores of TMT-B were positively correlated with PRS-SCZ in DS. Thus, the findings suggested that impaired executive function of the brain may be related to the abnormal state of DMN which was caused by the weakened strength of FC between INS and ANG.

Several limitations should be mentioned regarding this study. Firstly, in order to reduce the differentiation of the participating samples and the interference caused by social factors such as age, education, and gender, only male patients with chronic SCZ were recruited. Secondly, the image data of all the samples were not collected, which reduced the persuasive effect of the results. Future research will use a larger sample size and collect the complete experimental data to improve the reliability of the experimental results. Thirdly, it should be noted that this study did not successfully mitigate the influence of medication. The administration of medication might induce alterations in the functional characteristics of the brain. Subsequent studies should expand the scope by recruiting treatment-naïve schizophrenia patients as the primary cohort. Although the findings demonstrated imaging markers associated with PRS-SCZ in DS, further studies are warranted to clarify the specific mechanism. The genetic information acquisition for the samples in this paper was performed through whole-exome sequencing, potentially leading to the utilization of a limited number of SNPs in calculating PRS-SCZ. Subsequent studies will incorporate gene sequencing to ensure a more comprehensive gathering of genetic information.

In conclusion, the study demonstrated that DS own the lowest PRS-SCZ, indicating a lower genetic risk of SCZ. The correlation between altered resting-state fMRI features and PRS-SCZ in DS, without undergoing rigorous correction, may suggest a neuroimage-genetics perspective on the pathogenesis of DS.

### Supplementary information

Supplementary

## Data Availability

The data that support the findings of this study are available from the corresponding author, upon reasonable request.
