# Supplementary material for: Polygenic effects on brain functional endophenotype for deficit and non-deficit schizophrenia
Source: Schizophrenia (Heidelb). 2024 Feb 16;10(1):18. doi: 10.1038/s41537-024-00432-w (PMC10873412; doi:10.1038/s41537-024-00432-w)
Supplement: Supplementary file 1 — Supplementary [file 41537_2024_432_MOESM1_ESM.docx]

**Table S1**

| Demographic, cognitive and biochemical data of DS, NDS and HC. | | | | | |
| --- | --- | --- | --- | --- | --- |
| **Variable** | **DS** | **NDS** | **HC** | ***F/t*** | ***P*** |
|  | **（n=33）** | **(n=47)** | **(n=41)** |  |  |
| Age (years) | 50.94±8.11 | 51.91±7.96 | 51.85±6.54 | 0.189 | 0.828 |
| Education (years) | 8.98±2.192 | 9.28±1.486 | 9.83±2.167 | 1.967 | 0.144 |
| Age at Onset (years) | 22.55±3.71 | 22.81±3.96 |  | 0.09 | 0.765 |
| Duration of illness (years) | 28.39±7.52 | 29.11±7.85 |  | 0.165 | 0.686 |
| CPZ-equivalent daily dose (mg/day) | 480±231.44 | 572.02±215.10 |  | -1.810 | 0.074 |
| BPRS | 31.84±3.40 | 26.21±2.08 |  | 84.247 | <.001^#^ |
| **Sustained Attention** |  |  |  |  |  |
| TMT-A (s) | 118.89±57.48^*, #^ | 95.42±37.71^*^ | 57.63±16.14 | 23.318 | <.001 |
| Stroop words only | 43.58±19.97^*^ | 50.91±19.18^*^ | 71.17±15.01 | 23.998 | <.001 |
| Stroop colors only | 26.48±12.01^*, #^ | 32.49±12.33^*^ | 46.00±10.04 | 28.771 | <.001 |
| **Ideation Fluency** |  |  |  |  |  |
| COWAT | 2.79±2.88^*^ | 2.53±3.41^*^ | 11.78±4.49 | 83.178 | <.001 |
| **Cognitive Flexibility** |  |  |  |  |  |
| TMT-B (s) | 304.73±172.32^*^ | 257.53±103.82^*^ | 157.30±49.46 | 16.494 | <.001 |
| Stroop interference | 16.09±8.45^*, #^ | 21.28±9.26^*^ | 27.22±5.97 | 17.66 | <.001 |
| **Visuospatial Memory** |  |  |  |  |  |
| Spatial processing (block design) | 10.55±3.87^*, #^ | 12.60±3.71^*^ | 16.88±3.55 | 28.932 | <.001 |
| **Biochemical index** |  |  |  |  |  |
| TG | 1.60 ± 0.72 | 1.99 ± 1.15 |  | 2.963 | 0.089 |
| CHO | 4.35 ± 0.70 | 4.69 ± 0.93 |  | 3.161 | 0.079 |
| HDL | 1.06 ± 0.32 | 1.00 ± 0.25 |  | 0.935 | 0.336 |
| LDL | 2.59 ± 0.57 | 2.83 ± 0.66 |  | 2.769 | 0.1 |
| GLU | 5.51 ± 1.28 | 5.95 ± 1.27 |  | 2.263 | 0.137 |
| BDNF | 3.08 ± 1.78 | 3.57 ± 1.33 |  | 2.026 | 0.159 |
| GDNF | 522.51 ± 102.67 | 558.33±124.01 |  | 1.857 | 0.177 |

Notes: **Table** **S1** shows the sample with both genetic and imaging data, including their scores on the neurological assessments. Data is the mean ± SD. DS is the deficit schizophrenia group, NDS is the group of non-deficit schizophrenia patients and HC is the healthy control group. Post-hoc comparisons used the least-significant difference (LSD). TG: triglyceride, CHO: total cholesterol, HDL: high-density lipoprotein, LDL: low-density lipoprotein, BDNF: brain-derived neurotrophic factor, GDNF: glial cell line-derived neurotrophic factor. * Represents a comparison between a patient group and a healthy control group. * *p* < 0.05. ^#^ Represents a comparison between patient groups. ^#^ *p* < 0.05.

**Table S2**

The number of SNPS under different P threshold.

| P threshold | R^2^ | P | Num_SNP |
| --- | --- | --- | --- |
| 0.05 | 0.00831952 | 0.166083 | 28 |
| 0.1 | 0.012815 | 0.086531 | 40 |
| 0.2 | 0.011682 | 0.100635 | 69 |
| 0.3 | 0.0170213 | 0.047382 | 91 |
| 0.3137 | 0.0176141 | 0.043744 | 95 |
| 0.4 | 0.0114306 | 0.102732 | 114 |
| 0.5 | 0.0126647 | 0.086152 | 138 |
| 1 | 0.013289 | 0.078713 | 203 |

Notes: **Table** **S2** shows the number of SNPs included in the calculation

of polygenic risk scores for schizophrenia at different P thresholds, and

also shows the fit of the polygenic risk scores calculated for different

numbers of SNPs to the sample (R2).

**Table S3**

| Significant difference regions of FC based on the ROI analysis between DS and NDS (Seed Points: Left ITG). | | | | | |
| --- | --- | --- | --- | --- | --- |
| ROI Seed Points | Regions | Peak MNI Coordinates | | | Cluster Size |
|  | （DS vs NDS） | x | y | z |  |
| Left ITG | Right Corpus Callosm | 9 | -30 | 18 | 84 |
|  | Right Precuneus | 3 | -54 | 45 | 95 |
|  | Right Superior Temporal Gyrus | 60 | -48 | 9 | 177 |

Note: MNI, Montreal Neurological Institute; ITG, Inferior Temporal Gyrus.

**Table S4**

| Significant difference regions of FC based on the ROI analysis between DS and NDS (Seed Points: Left IFG). | | | | | |
| --- | --- | --- | --- | --- | --- |
| ROI Seed Points | Regions | Peak MNI Coordinates | | | Cluster Size |
|  | （DS vs NDS） | x | y | z |  |
| Left IFG | None | 0 | 0 | 0 | 0 |

Note: MNI, Montreal Neurological Institute; IFG, Inferior Frontal Gyrus.

**Table S5**

| Significant difference regions of FC based on the ROI analysis between DS and NDS (Seed Points: Right PCUN). | | | | | |
| --- | --- | --- | --- | --- | --- |
| ROI Seed Points | Regions | Peak MNI Coordinates | | | Cluster Size |
|  | （DS vs NDS） | x | y | z |  |
| Right PCUN | Right Cingulate Gyrus | 15 | -48 | 30 | 177 |
|  | Right Insula | 30 | 27 | 3 | 191 |
|  | Right Middle Frontal Gyrus | 33 | 33 | 18 | 114 |

Note: MNI, Montreal Neurological Institute; PCUN, Precuneus.

**Table S6**

| Significant difference regions of FC based on the ROI analysis between DS and NDS (Seed Points: Right MFG). | | | | | |
| --- | --- | --- | --- | --- | --- |
| ROI Seed Points | Regions | Peak MNI Coordinates | | | Cluster Size |
|  | （DS vs NDS） | x | y | z |  |
| Right MFG | Right Inferior Frontal Gyrus | 42 | -24 | 24 | 372 |

Note: MNI, Montreal Neurological Institute; MFG, Middle Frontal Gyrus.

**Table S7**

| Significant difference regions of FC based on the ROI analysis between DS and NDS (Seed Points: Right PoCG). | | | | | |
| --- | --- | --- | --- | --- | --- |
| ROI Seed Points | Regions | Peak MNI Coordinates | | | Cluster Size |
|  | （DS vs NDS） | x | y | z |  |
| Right PoCG | Left medial Superior Frontal Gyrus | -3 | 45 | 36 | 240 |
|  | Left Thalamus | -21 | -24 | 9 | 151 |

Note: MNI, Montreal Neurological Institute; PoCG, Postcentral Gyrus.

**Table S8**

| Significant difference regions of FC based on the ROI analysis between DS and NDS (Seed Points: Right ROL). | | | | | |
| --- | --- | --- | --- | --- | --- |
| ROI Seed Points | Regions | Peak MNI Coordinates | | | Cluster Size |
|  | （DS vs NDS） | x | y | z |  |
| Right ROL | Left Middle Frontal Gyrus | -36 | 42 | 30 | 146 |
|  | Right Middle Frontal Gyrus | 42 | 42 | 9 | 201 |
|  | Right Cingulate Gyrus | 12 | 15 | 39 | 339 |
|  | Left Angular | -33 | -54 | 30 | 166 |

Note: MNI, Montreal Neurological Institute; ROL, Rolandic operculum.

**Table S9**

| Significant difference regions of FC based on the ROI analysis between DS and NDS (Seed Points: Left INS). | | | | | |
| --- | --- | --- | --- | --- | --- |
| ROI Seed Points | Regions | Peak MNI Coordinates | | | Cluster Size |
|  | （DS vs NDS） | x | y | z |  |
| Left INS | Right Middle Frontal Gyrus | 33 | 45 | 9 | 155 |
|  | Left Middle Frontal Gyrus | -33 | 45 | 36 | 160 |
|  | Left Angular Gyrus | -48 | -63 | 33 | 142 |
|  | Left Cingulate Gyrus | -6 | 12 | 36 | 134 |

Note: MNI, Montreal Neurological Institute; INS, Insula.

**Table S10**

| Significant difference regions of FC based on the ROI analysis between DS and NDS (Seed Points: Right INS). | | | | | |
| --- | --- | --- | --- | --- | --- |
| ROI Seed Points | Regions | Peak MNI Coordinates | | | Cluster Size |
|  | （DS vs NDS） | x | y | z |  |
| Right INS | Right Middle Frontal Gyrus | 51 | 33 | 6 | 137 |
|  | Right Angular Gyrus | 60 | -54 | 27 | 139 |
|  | Left Angular Gyrus | -45 | -66 | 33 | 163 |
|  | Left Precuneus | -9 | -48 | 33 | 110 |

Note: MNI, Montreal Neurological Institute; INS, Insula.

**Figure S1**


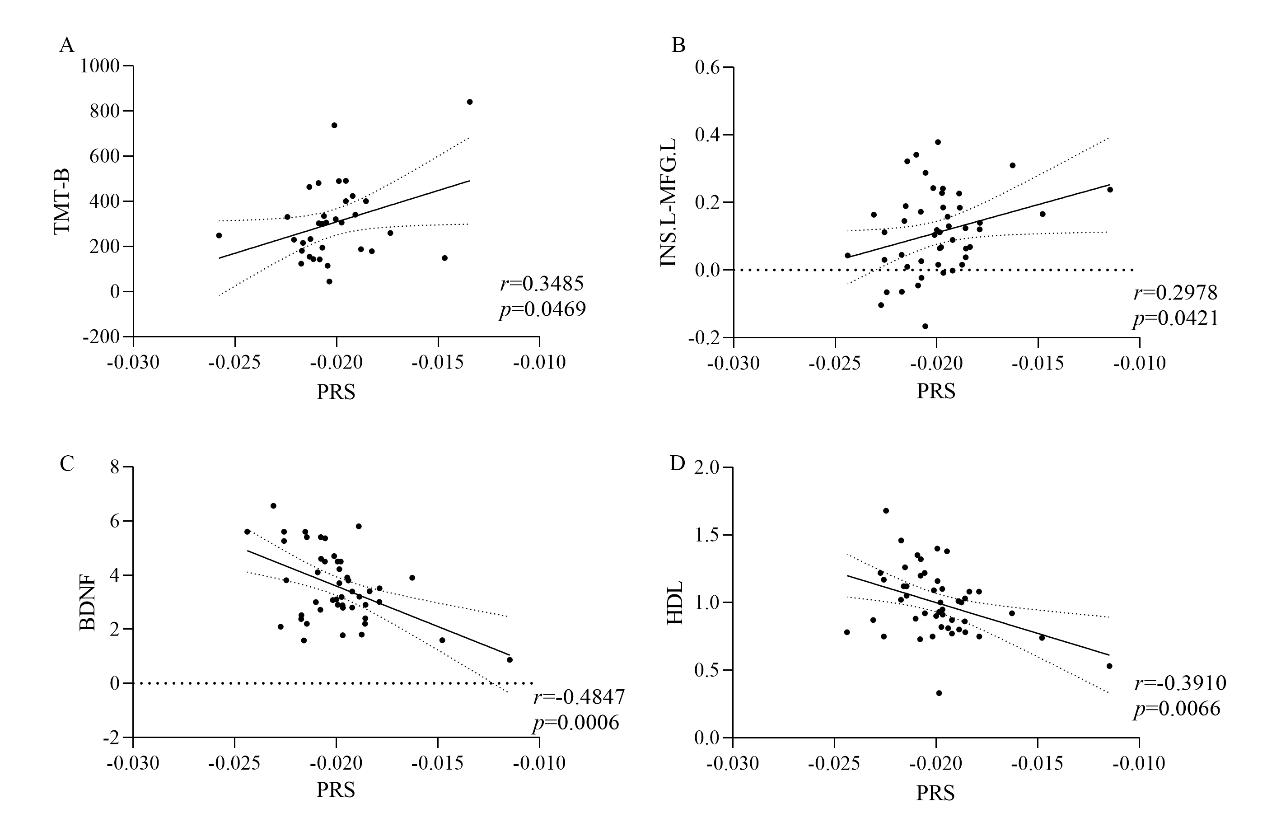


**Fig. S1 The associations of PRS-SCZ and significant (p < 0.05, uncorrected) correlation indices in DS and NDS groups.** All graphs take PRS-SCZ as the *x*-axis and each related variable as the *y*-axis. A: the scores was significantly correlated with PRS-SCZ in DS. B: the strength of functional connection between the left INS and the left MFG was significantly correlated with PRS-SCZ in NDS. C: the blood concentration of BDNF was significantly correlated with PRS-SCZ in NDS. D: the blood concentration of HDL was significantly correlated with PRS-SCZ in NDS.

**Figure S2**

**
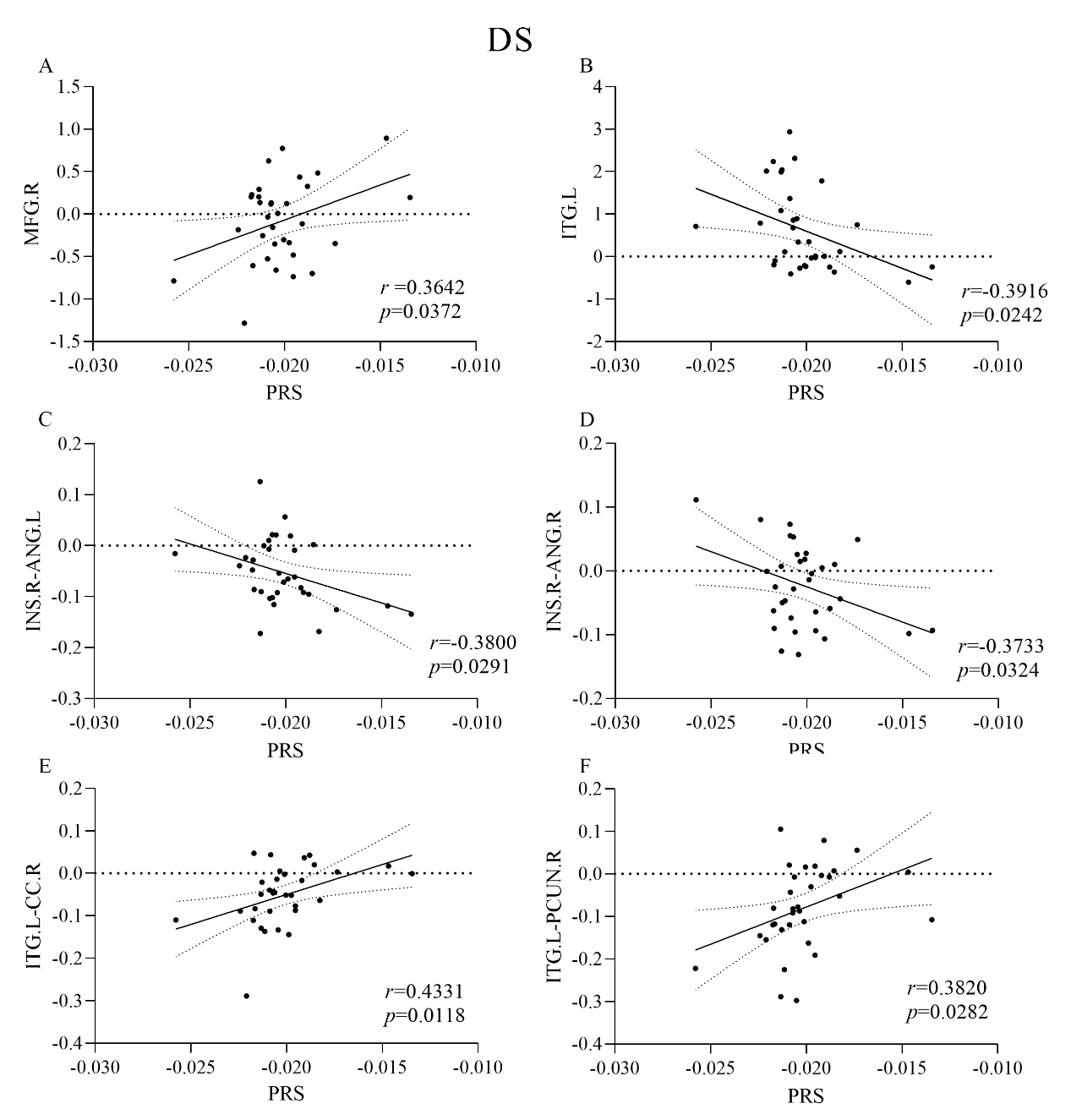
**

**Fig. S2 The associations of PRS-SCZ and each observable (****p < 0.05, uncorrected) correlation index in DS group.** All graphs take PRS-SCZ as the *x*-axis and each related variable as the *y*-axis. DS: deficit schizophrenia.
